# Minocycline Inhibition of Monocyte Activation Correlates with Neuronal Protection in SIV NeuroAIDS

**DOI:** 10.1371/journal.pone.0018688

**Published:** 2011-04-06

**Authors:** Jennifer H. Campbell, Tricia H. Burdo, Patrick Autissier, Jeffrey P. Bombardier, Susan V. Westmoreland, Caroline Soulas, R. Gilberto González, Eva-Maria Ratai, Kenneth C. Williams

**Affiliations:** 1 Department of Biology, Boston College, Chestnut Hill, Massachusetts, United States of America; 2 Harvard Medical School, Boston, Massachusetts, United States of America; 3 New England Regional Primate Research Center, Southborough, Massachusetts, United States of America; 4 Athinoula A. Martinos Center for Biomedical Imaging and Department of Radiology, Massachusetts General Hospital, Boston, Massachusetts, United States of America; Massachusetts General Hospital, United States of America

## Abstract

**Background:**

Minocycline is a tetracycline antibiotic that has been proposed as a potential conjunctive therapy for HIV-1 associated cognitive disorders. Precise mechanism(s) of minocycline's functions are not well defined.

**Methods:**

Fourteen rhesus macaques were SIV infected and neuronal metabolites measured by proton magnetic resonance spectroscopy (^1^H MRS). Seven received minocycline (4 mg/kg) daily starting at day 28 post-infection (pi). Monocyte expansion and activation were assessed by flow cytometry, cell traffic to lymph nodes, CD16 regulation, viral replication, and cytokine production were studied.

**Results:**

Minocycline treatment decreased plasma virus and pro-inflammatory CD14+CD16+ and CD14^lo^CD16+ monocytes, and reduced their expression of CD11b, CD163, CD64, CCR2 and HLA-DR. There was reduced recruitment of monocyte/macrophages and productively infected cells in axillary lymph nodes. There was an inverse correlation between brain NAA/Cr (neuronal injury) and circulating CD14+CD16+ and CD14^lo^CD16+ monocytes. Minocycline treatment *in vitro* reduced SIV replication CD16 expression on activated CD14+CD16+ monocytes, and IL-6 production by monocytes following LPS stimulation.

**Conclusion:**

Neuroprotective effects of minocycline are due in part to reduction of activated monocytes, monocyte traffic. Mechanisms for these effects include CD16 regulation, reduced viral replication, and inhibited immune activation.

## Introduction

Human immunodeficiency virus (HIV) infection of the central nervous system (CNS) can result in cognitive impairment, behavioral deficits, and motor dysfunction. With the use of anti-retroviral therapy (ART) the incidence of HIV-associated neurological disease has declined [1]. While ART prolongs health and longevity of HIV-infected individuals, the majority of anti-retroviral drugs have poor CNS penetration. As a result, the prevalence of neurologic complications in HIV-infected patients continues to rise [2]. Factors mediating inflammatory responses outside the CNS likely play critical roles in CNS dysfunction. Monocyte/macrophage traffic likely plays a significant role in driving CNS neuropathogenesis [3]–[6].

Monocyte traffic across the blood-brain barrier (BBB) occurs at a basal level that increases with immune activation [7]. Such traffic likely serves as a primary route of viral entry into the CNS [8] and regulates the accumulation of macrophages in encephalitic lesions, which are the histopathological correlate of HIV-associated neurocognitive disorders (HAND). The majority of monocytes express the lipopolysaccharide (LPS) receptor CD14, while only approximately ten percent also express the FcγIII receptor CD16 under normal conditions [9], [10]. Following viral infection, with inflammation, the number of monocytes as well as the percentages of activated monocyte subsets increase, resulting in increased traffic to and accumulation within tissues including the brain [11], [12]. Once activated, CD14+CD16+ and CD14^lo^CD16+ monocytes express high levels of pro-inflammatory cytokines that are linked to the development of HAND and simian immunodeficiency virus encephalitis (SIVE) [12], [13]. With HIV and SIV infection, the number of CD14+CD16+ monocytes increases [14], [15]. HIV and SIV DNA and RNA are found in both CD14+CD16− and CD14+CD16+ monocyte subsets in acute infection and AIDS. Viral DNA is consistently found in CD14+CD16+ monocytes throughout disease [16], [17]. We have shown that perivascular macrophages are repopulated from bone marrow in normal rhesus macaques [18] and are a primary cell productively HIV and SIV infected in the CNS [19], [20]. Populations of monocytes are immunophenotypically similar to CNS perivascular macrophage; both express CD14, CD16, and CD163. Thus, it is likely that subsets of CD14+CD16+CD163+ monocytes, some of which are infected, repopulate CNS perivascular macrophages [6], [21]. Thus, therapies targeting monocyte/macrophages outside the CNS can potentially affect neuronal injury.

Minocycline, a lipid soluble tetracycline antibiotic that has putative effects on immune system cells, fortuitously can also effectively cross the blood brain barrier (BBB) into the CNS parenchyma [22]. Several studies established that minocycline possesses anti-inflammatory and possibly direct neuroprotective properties independent of its antimicrobial effects [23], [24]. Animal studies indicate minocycline inhibits the production of immune activators by macrophages, microglia [25]–[28], and neurons [26]–[28]. Minocycline inhibits activation, proliferation, and viral replication of microglia, macrophages, and lymphocytes *in vitro* [25], [28]–[30]. In SIV-infected pigtailed macaques, minocycline reduced plasma virus, the pro-inflammatory monocyte chemoattractant protein 1 (MCP-1)/CCL2, and viral DNA in the CNS [28]. Whether decreased monocyte/macrophage activation by minocycline also plays a neuroprotective role via such mechanisms is not well-defined. To date studies correlating neuronal injury simultaneously with viral infection and monocyte/macrophage activation have not been done.

Here, we report the effects of minocycline on monocyte/macrophage numbers and activation, and neuronal injury in a pathogenesis study. We used a CD8+ T lymphocyte depletion model of SIV infection in rhesus macaques, which results in rapid progression to AIDS (3–4 months) with a high incidence of SIVE [31]. Using this model and magnetic resonance (MR) spectroscopy we found that minocycline treatment resulted in stable N-acetylaspartate to Creatine (NAA/Cr) levels in the brain (representing neuronal protection) compared to non-treated animals, which continued to decline (consistent with neuronal injury) [32]. In the current study, using the same cohort and three additional control non-minocycline treated animals, we report minocycline treatment reduced activation of monocytes that inversely correlated with neuronal injury, reduced the accumulation of monocyte/macrophages in lymph nodes of treated animals, and inhibited the expression of CCR2, CD163, CD11b, and CD64 on monocytes. These results suggest that minocycline, by down-regulating CD16 and viral replication, inhibiting monocyte activation and immune cell traffic, is neuroprotective.

## Results

Fourteen animals were SIV-infected and treated with an anti-CD8+ T lymphocyte antibody (cM-T807), administered at 6, 8, and 12 days post infection (dpi). Three were transiently CD8+ lymphocyte depleted (≤21 dpi), while the remaining eleven were persistently CD8+ lymphocyte depleted (>28 dpi) (Table 1). Over the course of the study, there were no significant differences in the plasma viral load or numbers of monocyte subsets between the transiently and persistently CD8 lymphocyte depleted animals. We have previously shown that persistent CD8+ lymphocyte depletion results in rapid AIDS (3–4 months) with a high incidence of SIVE (>85%) [33], [34]. Minocycline (4 mg/kg/day) was initiated 28 dpi given daily as we previously reported [32]. Animals were sacrificed with the development of AIDS or at a previously determined timed sacrifice. Plasma SIV RNA peaked at 10^8^ copy eq./mL by 12 dpi. Plasma virus decreased by approximately one log after 7 days of minocycline treatment and remained at that level until sacrifice (Table 1).

**Table 1:** SIV-infected, CD8+ T Lymphocyte depleted animals used in this study.

Animal no.	Minocycline	Length of infection (days)	CD8+ lymphocyte depletion^a^	Terminal plasma viral load (copy eq. /mL)^b^
74 – 05	None	56	Persistently depleted	5.6×10^7^
79 - 05	None	42*	Persistently depleted	6.3×10^7^
156 - 04	None	62	Persistently depleted	3.6×10^8^
346 - 04	None	62	Persistently depleted	6.2×10^8^
307 - 05	None	43*	Persistently depleted	6.5×10^7^
118 - 07	None	57	Persistently depleted	1.8×10^8^
121 - 07	None	57	Persistently depleted	2.2×10^8^
94 - 04	Started 28 dpi	60	Persistently depleted	9.0×10^6^
35 - 07	Started 28 dpi	62	Persistently depleted	8.0×10^7^
150 - 04	Started 28 dpi	60	Persistently depleted	8.3×10^7^
150 - 05	Started 28 dpi	55	Persistently depleted	6.3×10^7^
48 - 07	Started 28 dpi	62	Transiently depleted	4.3×10^6^
227 - 04	Started 28 dpi	60	Transiently depleted	8.9×10^6^
258 - 04	Started 28 dpi	62	Transiently depleted	2.7×10^7^

Note.-dpi = days post infection.

*Untreated animals time-sacrificed at 6 weeks pi; all other animals sacrificed at 8 weeks pi.

a Transiently CD8+ lymphocyte depleted (≤21 dpi), persistently CD8+ lymphocyte depleted (>28 dpi).

b Viral RNA quantitiated using RT PCR and are results of duplicate measurements.

**Figure 1:**
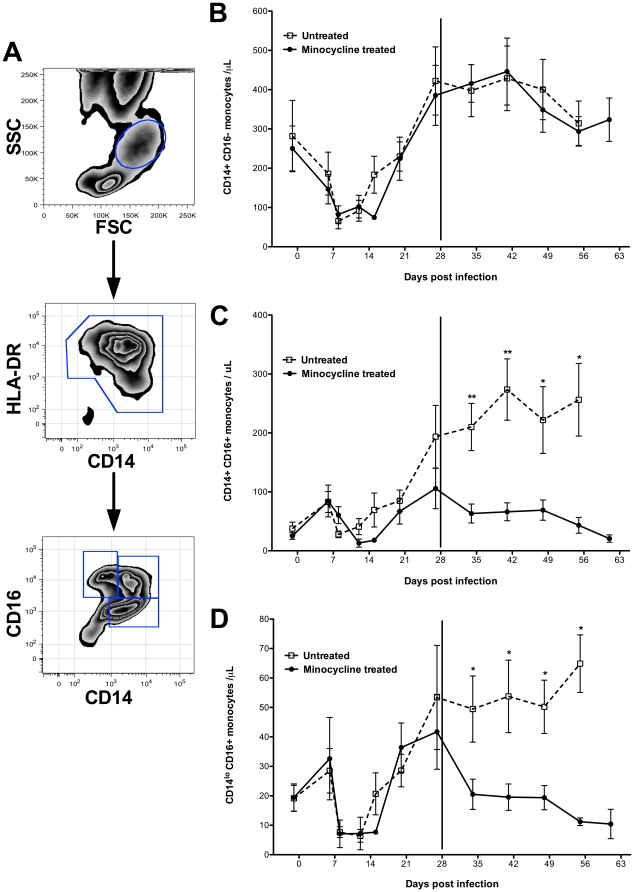
Minocycline reduces expansion of activated monocytes. Flow cytometric analysis of CD14+CD16−, CD14+CD16+, and CD14^lo^CD16+ monocyte populations. Using flow cytometric analyses, monocytes were first selected based on size and granularity (FSC vs. SSC). From this gate, HLA-DR+ CD14+ monocytes were selected. A small population of CD14-negative, HLA-DR-negative cells, likely representing lymphocytes or dendritic cells, were excluded. We note that all monocytes are HLA-DR+ based on FSC vs. SSC and that the number of HLA-DR+ and absolute number of monocytes are equivalent. From the CD14+ HLA-DR+ gate monocyte subsets were fractionated based on CD14 and CD16 expression (**A**). Comparisons were made between SIV-infected, CD8+ T lymphocyte depleted animals with (filled symbols) and without (open symbols) minocyline treatment. Minocycline treatment was initiated at day 28 post-infection (start date is marked by a solid vertical line). The absolute number of CD14+CD16− monocytes was comparable between groups throughout the course of the study (**B**). In contrast, numbers of CD14+CD16+ (**C**) and CD14^lo^CD16+ (**D**) monocytes were significantly higher in untreated animals at all points compared to minocycline treated animals. Data points represent the mean ± standard error of the mean (n = 7 animals per group until 6 weeks pi when two untreated animals were time-sacrificed). *P* values were calculated using a Mann-Whitney U test (*p*<0.05*, *p*<0.01**).

Flow cytometric analyses were completed by employing a gating strategy where peripheral blood monocytes were initially identified according to forward scatter and side scatter properties (Figure 1A) [10], [34]. A small population of CD14-negative, HLA-DR-negative cells, likely representing lymphocytes or dendritic cells, was excluded by gating on all CD14+ HLA-DR+ cells. Within this gate, monocyte subsets were defined by expression of CD14 versus CD16. The absolute numbers of classical CD14+CD16− monocytes were comparable between groups prior to and after minocycline treatment (Figure 1B). The absolute number of activated CD14+CD16+ (Figure 1C) and CD14^lo^CD16+ (Figure 1D) monocytes increased in the untreated group but were significantly reduced in minocycline treated animals at all time points.

The median fluorescence intensity (MFI) of CD11b, CD163, CCR2, CD64 and HLA-DR on monocyte subsets prior to minocycline treatment (day 27) and terminally was examined (Table 2). Day 27 was selected because it was a time point immediately preceding minocycline treatment and is a point of peak monocyte activation. All markers studied on monocyte subsets from SIV-infected macaques without minocycline treatment were increased one to four-fold at sacrifice compared to day 27 pi. In contrast, there was a two to seven-fold decrease in MFI expression of CD11b, CD163, CCR2, CD64, and HLA-DR between minocycline treated and untreated animals terminally. Interestingly, the MFI of these markers on monocytes from minocycline treated animals terminally was very similar to the values found the prior to treatment indicating that minocycline treatment reduces monocyte/macrophage activation *in vivo*. Additionally, the MFI for HLA-DR decreased on all monocyte subsets two-fold less than the pretreatment values (Table 2). Thus, minocycline treatment reduces the expression of several markers critical for monocyte traffic and function at late stage of infection, and in the case of HLA-DR is reduced below that found prior to minocycline treatment.

**Table 2:** Activation Markers on Monocyte Subsets.

Monocyte Subset	Day 27 (MFI)	Terminal (MFI)		*P* value
	All Animals	Untreated	MN Treated	
CD14+CD16−				
CD11b	11085 (5382)	37149 (17100)	10425 (1603)	*
CD163	3178 (823)	8791 (2600)	3651 (1911)	*
CCR2	478 (25)	1971 (649)	463 (170)	ns
CD64	3443 (201)	5746 (842)	3793 (357)	*
HLA-DR	3964 (950)	5480 (1747)	1754 (972)	*
CD14+CD16+				
CD11b	11099 (5261)	37560 (16295)	10171 (1184)	***
CD163	4156 (1047)	9140 (2381)	3728 (1754)	*
CCR2	182 (58)	1172 (306)	303 (109)	ns
CD64	1209 (162)	2175 (419)	1435 (96)	*
HLA-DR	8060 (1875)	13160 (3689)	4145 (2456)	*
CD14^lo^CD16+				
CD11b	2715 (1131)	6807 (2751)	1822 (483)	*
CD163	1344 (340)	3328 (572)	1214 (642)	*
CCR2	27 (92)	360 (115)	113 (25)	ns
CD64	390 (20)	763 (206)	434 (179)	ns
HLA-DR	6010 (1408)	9247 (3465)	1227 (186)	*

Note.-Means are the median fluorescence intensity, MFI, and the SEM is in brackets.

MFI were calculated by subtracting the MFI of the appropriate isotype controls.

P values were calculated by comparing terminal MFI values for untreated and minocycline treated animals using a Mann-Whitney U test (*p*<0.05*, *p*<0.001***). MN = minocycline.

**Figure 2:**
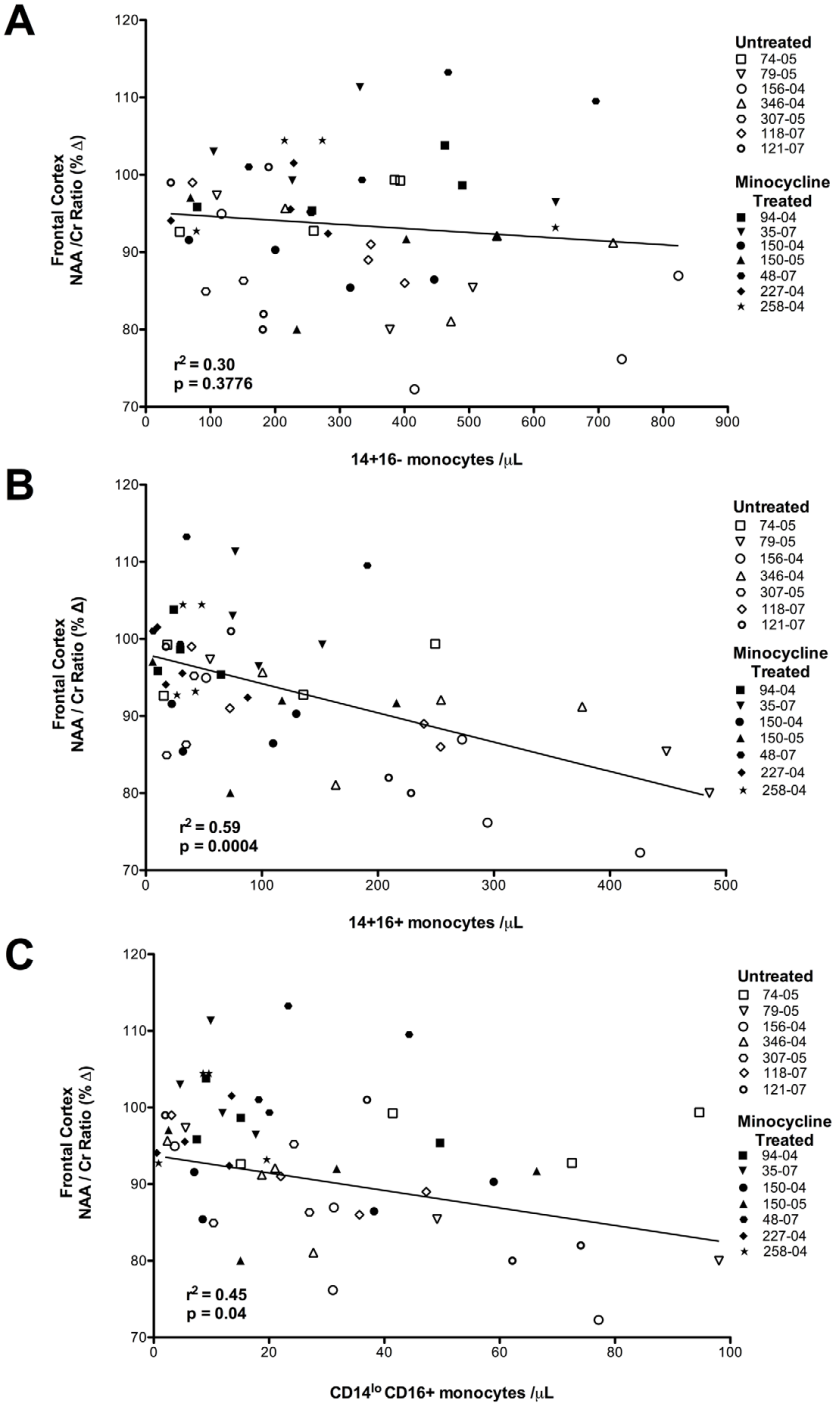
Linear regression analyses reveal significant relationships between circulating pro-inflammatory monocytes and NAA/Cr. Regression analyses were performed between the absolute numbers of each monocyte subset and the percent change in neuronal metabolite values (NAA/Cr) in the frontal cortex relative to pre-infection levels for all animals from 28 days pi until necropsy. Minocycline treated and non-treated animals were examined at 4 time-points except for the two animals that were time-sacrificed at 6 weeks pi, have three time-points. We found a significant inverse relationship between both CD14+CD16+ (**B**; r^2^ = 0.59, *p* = 0.0004) and CD14^lo^CD16+ (**C**; r^2^ = 0.45, *p* = 0.04) monocytes and decreased NAA/Cr, while no relationship between CD14+CD16− monocytes and NAA/Cr levels were observed (**A**; r^2^ = 0.30, *p* = 0.3776).

Next, we examined possible correlations between longitudinal changes in monocyte numbers and the ratio of N-acetylaspartate to Creatine (NAA/Cr) in different brain regions of the same animals with and without minocycline treatment. The NAA/Cr ratio in the frontal cortex, parietal cortex, white matter, and basal ganglia was previously determined and reported [32] where decreases were found in all SIV-infected animals prior to minocycline treatment (dpi 27) [32]. At four weeks pi, following minocycline treatment, NAA/Cr in treated animals was stabilized, whereas untreated animals had a continued decline [32]. Linear regression analyses revealed a highly significant relationship between the absolute number of pro-inflammatory CD14+CD16+ and CD14^lo^CD16+ monocytes and NAA/Cr (in all brain regions). The relationship between monocyte subsets and NAA/Cr in the frontal cortex (CD14+CD16+: r^2^ = 0.59, *p* = 0.0004; CD14^lo^CD16+: r^2^ = 0.45, *p* = 0.04) representative of the other brain regions is illustrated in Figure 2. In the parietal cortex we found: CD14+CD16+: r^2^ = 0.67, *p* = 0.0003; CD14^lo^CD16+: r^2^ = 0.55, *p* = 0.0007. In the basal ganglia we found: CD14+CD16+: r^2^ = 0.53, *p* = 0.039; CD14^lo^CD16+: r^2^ = 0.26, *p* = 0.36. In the white matter: CD14+CD16+: r^2^ = 0.52, *p* = 0.0012; CD14^lo^CD16+: r^2^ = 0.50, *p* = 0.0006. We only found a significant correlation between CD14+CD16− monocytes and NAA/Cr in the parietal cortex (CD14+CD16−: r^2^ = 0.36, *p* = 0.02). Correlations between CD14+CD16− monocytes in other brain regions were not significant (data not shown). The inverse relationship between activated CD14+CD16+ and CD14^lo^CD16+ monocytes with NAA/Cr coupled with the observation that minocycline treatment reduces the number of activated monocytes, supports the notion that there is a link between alterations of NAA/Cr and the number of activated monocytes.

**Figure 3:**
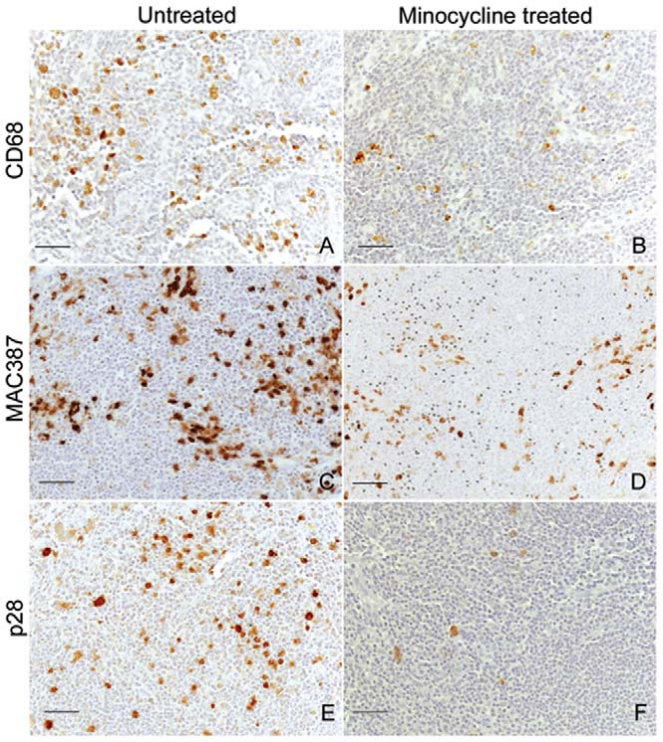
Reduced CD68+, MAC387+, and SIV p28+ productively infected cells in lymph nodes with minocycline treatment. Immunohistochemistry was performed to compare CD68+ resident macrophages, newly infiltrating MAC387+ monocytes/macrophages, and SIV-infected cells in the axillary lymph node from untreated and minocycline treated animals. A reduced number of resident CD68+ macrophages (**A**–**B**) and newly infiltrating MAC387+ monocytes/macrophages (**C**–**D**) in an axillary lymph node of minocycline treated animals (**B**, **D**) and an untreated controls (**A**, **C**). In addition, there was a significantly higher number of productively infected SIV p28+ cells in the lymph node of untreated animals (**E**) compared to minocycline treated animals (**F**). All scale bars are 50 µm.

**Figure 4:**
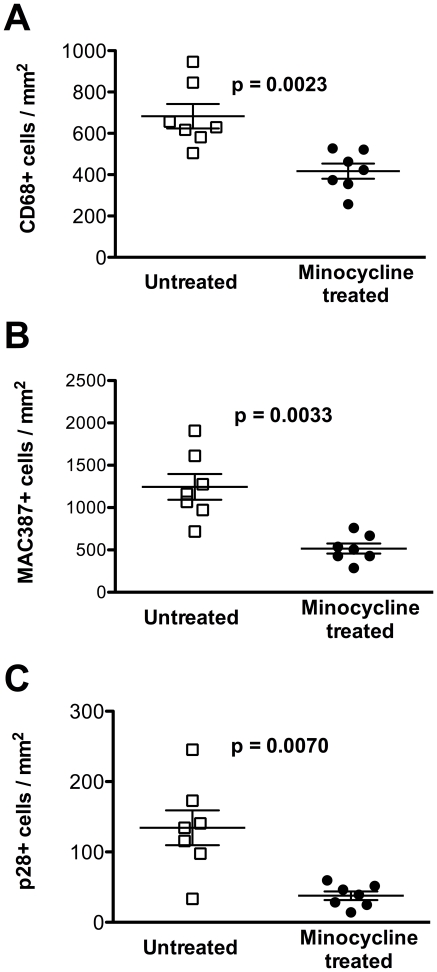
Minocycline reduces the number of CD68+, MAC387+, and p28+ cells in axillary lymph node. Quantitative analysis of CD68+ revealed significantly fewer CD68+ cells in the axillary lymph node of animals that received minocycline as compared to untreated controls (**A**; *p* = 0.0023); fewer numbers of newly infiltrating MAC387+monocytes/macrophages (**B**; *p* = 0.0033); and a decreased number of productively SIV infected p28+ cells (**C**; *p* = 0.0070). Numbers are representative of the means from a minimum of twelve fields calculated to represent a single data point for each animal. Horizontal bars indicate group mean values and error bars indicate the standard error of the mean. P values were determined using a Mann-Whitney U test.

We previously reported that none of the minocycline treated animals developed SIVE (defined as the accumulation of monocyte/macrophages, virally infected cells, and multi-nucleated giant cells) [32]. Examining axillary lymph nodes we found a statistically significant reduction in the relative numbers of resident mature CD68+ macrophages (Figure 3A–B, Figure 4A; *p* = 0.0023), recently recruited MAC387+ monocytes/macrophages (Figure 3C–D, Figure 4B; *p* = 0.0033), and productively infected SIV p28+ cells (Figure 3E–F, Figure 4C; *p* = 0.0070) with minocycline. This finding is consistent with reduced traffic and activation of monocyte/macrophages, as well as productive infection in lymph nodes (Figure 4A–C) similar to the decreased infection we reported in the CNS [32].

**Figure 5:**
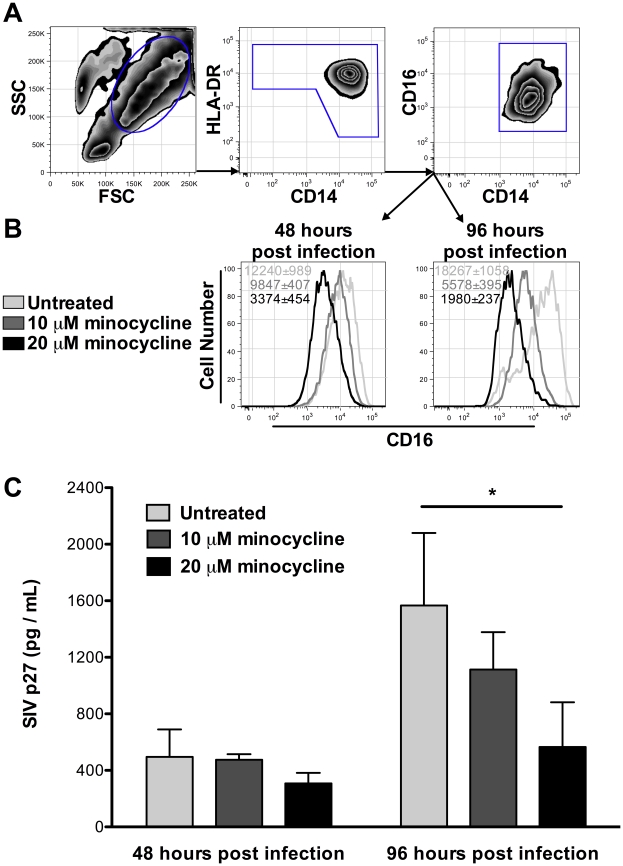
Reduction of CD16 expression and viral replication in CD14+ monocytes during *in vitro* minocycline treatment. CD14+ monocytes were infected with SIVmac316STOP virus and cultured with M-CSF in the presence or absence of minocycline for 24 and 72 hours. With M-CSF treatment, all monocytes expressed CD14 and CD16 prior to minocycline treatment. By flow cytometry, monocytes were first gated based on size (FSC) and granularity (SSC). From this gate HLA-DR+ CD14+ monocytes were selected (**A**) and CD16 expression on these cells between treatment groups was compared. Histograms represent the median fluorescence intensity (MFI) of CD16 from one representative experiment out of three (**B**). Averages of MFI ± standard error of the mean in a given treatment group are indicated in the upper left hand corner of the graphs. CD16 expression was significantly higher on untreated than 20 µM minocycline treated monocytes at 48 hours pi (*p* = 0.021). Untreated monocytes had significantly higher CD16 expression than both 10 µM and 20 µM treated cells at 96 hours pi (*p* = 0.001). After 96 hours of infection, SIV-p27 was reduced with minocycline treatment (**C**), with significant differences between control and 20 µM minocycline (*p* = 0.039). Studies presented here are the results of n = 3 three independent experiments with n = 3 animals per experiment performed in triplicate wells. P values were determined using a Mann-Whitney U test.

*In vitro* experiments were used to determine the effect of minocycline on monocyte CD16+ with viral infection. CD16 expression on monocyte/macrophages was reduced following10 µM minocycline for 24 hours, and was significantly reduced using 20 µM minocycline for 24 hours (Figure 5). By 72 hours of treatment, CD16 expression was significantly decreased on minocycline treated cells at both concentrations (Figure 5A–B) while CD14 expression was unchanged (data not shown). These data suggest that by down-regulating CD16, minocycline treatment may prevent differentiation, activation, or both on monocyte/macrophages. Such inhibition of monocyte/macrophage activation or differentiation *in vivo* may result in decreased replication or abundance of CD14+CD16+ target cells for HIV and SIV. In addition, 20 µM minocycline *in vitro* significantly reduced SIV replication by monocyte/macrophages 96 hours post-infection (Figure 5C). Whether the inhibition of viral replication in monocytes *in vitro* is due to a block of viral entry or post entry event requires further study. Minocycline did not result in monocyte cell death as measured by LIVE/DEAD cell staining (data not shown). We note that *in vitro* doses of minocycline used here (10 µM and 20 µM) are similar to those found in serum of minocycline treated humans [35]. Collectively, these data indicate that both CD16 expression and viral replication are reduced with minocycline treatment, consistent with the effects of minocycline observed *in vivo*.

**Figure 6:**
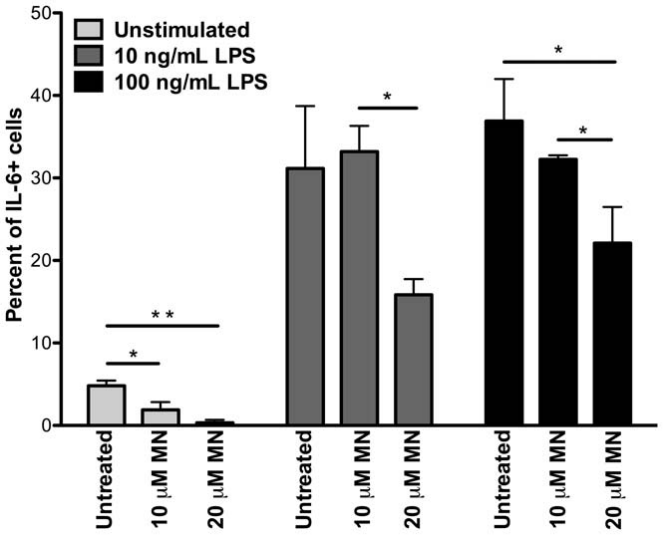
Minocycline treatment inhibits induction of IL-6 by CD14+ monocytes *in vitro*. CD14+ monocytes were cultured in non-adherent conditions with M-CSF and in the presence or absence of minocycline for 16 hours. IL-6 was induced by LPS in the presence of brefeldin A for 3 hours. The percentage of monocytes producing IL-6 was significantly lower with 10 µM (*p* = 0.04) and 20 µM (*p* = 0.009) minocycline than in untreated cells. With 10 ng/mL LPS, the percent of IL-6 producing monocytes was significantly reduced with 20 µM as compared to 10 µM minocycline treatment (*p* = 0.016). With 100 ng/mL LPS stimulation, minocycline treatment at both 10 µM (*p* = 0.058) and 20 µM (*p* = 0.03) doses significantly inhibited IL-6 cytokine secretion in CD14+ monocytes. Data presented represent the results of two independent experiments with n = 3 animals per experiment performed in triplicate wells. P values were determined using a Mann-Whitney U test.

Further *in vitro* experiments were completed to determine the effect of minocycline on pro-inflammatory cytokine production in response to LPS stimulation (Figure 6). The percentage of CD14+ monocytes producing IL-6 after 3 hours of culture without stimulation was significantly lower with 10 µM (*p* = 0.04) and 20 µM (*p* = 0.009) minocycline treatment. Following 3 hours of stimulation with 10 ng/mL LPS, the percent of IL-6 producing monocytes was significantly lower with 20 µM than with 10 µM minocycline (*p* = 0.016). With a higher concentration of 100 ng/mL LPS, minocycline treatment at both 10 µM (*p* = 0.058) and 20 µM (*p* = 0.03) doses significantly inhibited IL-6 cytokine secretion in CD14+ monocytes (Figure 6). TNF production in response to LPS stimulation was also examined, however there was no significant difference between untreated and minocycline treated monocytes following 10 ng/mL or 100 ng/mL LPS (data not shown).

## Discussion

Here, we demonstrate a correlation between expansion of activated monocytes and neuronal protection with minocycline in a rapid model of SIV-neuropathogenesis. We found decreased traffic of monocyte/macrophages to lymph nodes in minocycline treated animals, and *in vitro* evidence of a down-regulation of CD16 expression, a marked decrease in viral replication, as well as inhibition of IL-6 production following LPS stimulation. In minocycline treated animals we did not observe an expansion of CD14+CD16+ and CD14^lo^CD16+ monocytes that was observed in untreated animals with AIDS. These data parallel our previous results showing a direct relationship between the expansion of activated monocyte populations and decreased NAA/Cr [32].

Although it is difficult to determine the exact timing of CNS pathological changes, neuronal injury as measured by decreased NAA/Cr was detected by two weeks pi coincident with an elevation in activated CD14+CD16+ monocytes. Virus enters the CNS consistently by two weeks pi likely through trafficking of monocytes into the brain [34], [36]. This appears to be sufficient for the induction of neuronal damage. We observed a decrease in the absolute number of monocytes with minocycline treatment with no further CNS damage, supporting the notion that monocyte expansion is required to drive disease.

In previous work, we observed a bi-phasic increase in the number and relative percentage of activated monocytes with the second peak occurring with peripheral immune system dysfunction and a steep decline in NAA/Cr ratios [33]. The levels of circulating monocytes in untreated animals followed this well-described pattern of biphasic monocyte expansion with the development of AIDS, but this second peak was not seen in the minocycline treated animals. In fact there was a decrease in the number of such cells as well as the level of immune activation and accessory molecules on total monocytes.

The neuroprotective effects of minocycline confirm previous observations by Zink et al. [28] who found decreased activation of tissue macrophages, CNS viral load, and severity of CNS disease with minocycline treatment in SIV-infected pigtail macaques. Our results extend these findings by demonstrating that the effects of minocycline are directly correlated with reduced number of activated monocyte/macrophage and decreased activation markers on these pro-inflammatory cells. Zink and colleagues also examined alterations in the potent monocyte chemoattractant protein (MCP-1) throughout infection and with minocycline treatment. MCP-1 concentration in CSF followed a biphasic pattern with elevations during acute infection that declined after 10–14 dpi and again increased after four weeks of infection. In macaques treated with minocycline during chronic infection, the second peak in MCP-1 levels in CSF was not observed [37]. This is very similar to our observations that minocycline treated macaques did not have a second wave of activated CD14+CD16+ and CD14^lo^CD16+ monocytes. In addition, although expression of the MCP-1 receptor CCR2 was also significantly increased on the pro-inflammatory monocytes of untreated animals in our study, we found CCR2 levels remained unchanged on activated monocytes from minocycline treated animals, suggesting that minocycline may disrupt the recruitment and trafficking of highly activated monocytes into the CNS. This was further supported by our findings of reduced recruitment, viral replication, and activation of macrophages in the lymph nodes of minocycline treated animals.

Classically activated CD14+CD16− monocytes express CD64 (FcγRI), CCR2, low levels of HLA-DR, and release cytokines such as IFN-β and IL-10 [38]. In response to inflammation and viral infection, there is an expansion of more mature CD14+CD16+ and CD14^lo^CD16+ monocyte populations first with acute infection and again with the development of AIDS, when these cells can represent up to 40% of the total circulating monocyte population [10], [33]. These monocytes express high levels of CD11b, CD163, and HLA-DR and release high levels of pro-inflammatory cytokines including TNF-α, IFN-γ, and IL-6 [19]. Interestingly we found minocycline significantly reduced the number of pro-inflammatory monocytes, but did not affect the number of classical CD14+CD16− monocytes. This may have resulted from suppressed activation and maturation of monocytes, down-regulation of CD16 *in vivo*, and/or decreased turnover and trafficking of this subset of myeloid cells from the bone marrow.

In addition to its anti-inflammatory effects, minocycline inhibits the growth of a wide variety of Gram-negative and Gram-positive bacteria. Following oral administration, concentrations of minocycline are highest in the bile and small intestine [39]. It is therefore conceivable that in our SIV infection model of rapid neuroAIDS, minocycline binds to and eliminates microbial products in the gut, resulting in decreased TLR4 signaling and inhibited expansion of highly activated CD16+ monocytes. In this study, we assayed for LPS in plasma, but did not find significant differences in levels of LPS between the treatment groups. This does not rule out that minocycline might directly effect the response of monocytes *in vivo* to translocated bacterial products. Decreased expression and therefore potentially crosslinking of Fcγ receptors (CD16 and CD64) by antibody opsonized microbes in minocycline treated monocytes could have resulted in decreased transcription of inflammatory genes [40]. Minocycline treatment has also been shown to strongly chelate iron, which is an essential nutrient required by bacteria to survive and multiply [41].

Recent studies by Szeto et al. [42] indicate that by suppressing lymphocyte activation, minocycline treatment reduces HIV replication in CD4+ T lymphocytes. We observed a similar effect in CD14+ monocytes *in vitro*, where reduction of viral replication was directly related to the extent of CD16+ expression. It is important to note that the concentrations of minocycline that we used *in vitro* are physiologically similar to those found in humans with minocycline treatment [35], [43]. These results indicate that the antiviral effects of minocycline are linked to its ability to reduce activation of monocytes and their permissiveness to viral infection. CD14+CD16+ and CD14^lo^CD16+ monocytes are considered to be at an advanced stage of maturation, and it has been proposed that these cells are preferentially infected and harbor viral particles long-term [16], [17], [44]. Current evidence suggests that restriction of viral replication in less mature CD14+CD16− monocytes is mediated by differentiation-dependent cofactors such as apolipoprotein B mRNA-editing enzyme 3G (APOBEC3G) and APOBEC3A [45]. Upon CD16-mediated activation, the transcriptional activators NF-κβ and C/EBPβ, which are essential factors for viral replication in monocytes, are also induced [46]. Based on the results from our study, it is conceivable that minocycline treated monocytes display a restriction to SIV replication similar to that of classically activated CD14+CD16− monocytes.

Despite the reported beneficial effects of minocycline in several animal models of CNS disease, including ALS [47]–[49] a recent clinical trial with ALS patients found patients deteriorated significantly faster than the placebo control group [50]. These results underscore that caution and more studies are required before additional clinical work with minocycline. In addition, this study underscores the importance of understanding differences between animal models of disease and disease. Our data support the notion that inhibition of monocyte/macrophage activation, and possibly viral infection, correlates with neuronal protection assessed by MRS. Our results suggest that minocycline may be beneficial as an adjunctive therapy, to antiretroviral therapies, that are less effective in crossing the BBB. This data was found using an SIV model of CNS neuroAIDS, which might more accurately mirror CNS pathology, than mouse models of ALS mirror the human disease.

We report here that suppression of chronic immune activation with minocycline treatment results in the reduced expansion of highly activated and potentially infected pro-inflammatory monocytes. Decreased expression of receptors such as CD11b, CD16, and CCR2 critical for trafficking of monocytes into the brain demonstrates that minocycline prevented the recruitment of these highly invasive cells into the CNS.

## Materials and Methods

### Ethical Treatment of Animals

These studies were performed with the approval of the Massachusetts General Hospital Subcommittee on Research and Animal Care and the Institutional Animal Care and Use Committee of Harvard University. Animals were housed according to the standards of the American Association for Accreditation of Laboratory Animal Care. Treatment of animals was in accordance with the Guide for the Care and Use of Laboratory Animals of the Institute of Laboratory Animal Resources.

### Animals, SIV infection, CD8+ T lymphocyte depletion, and Minocycline treatment

The cohort of animals used in this study was reported in a recent publication of the effects of minocycline on CNS neural metabolites using MR spectroscopy [32]. Three additional non-minocycline treated animals were also included in the experiments presented here for a total of n = 14 animals. In the current manuscript, we report the effects of minocycline on monocytes from animals in this cohort, and perform correlations of monocyte numbers vs. n-acetylaspartate/creatine (NAA/Cr), a marker of neuronal injury. Fourteen rhesus macaques (*Macaca mulatta*) were intravenously inoculated with SIVmac251 (20 ng SIV p27; a generous gift from Dr. Ronald Desrosiers, NERPC) as previously described [32]. CD8+ T lymphocyte depletion was achieved using cM-T807, an anti-CD8+ antibody that was administered subcutaneously (10 mg/kg) on day 6 post infection (pi) and intravenously (5 mg/kg) on days 8 and 12 pi [31], [51], [52]. Minocycline was orally administered twice daily (2 mg/kg) to seven animals beginning four weeks pi and continuing throughout the study [32]. Macaques were sacrificed upon development of AIDS or at a predetermined timed sacrifice following four weeks of minocycline treatment.

### Viral load, MRI and MRS

Plasma SIV RNA was quantified using real-time PCR as previously described [52]. NAA/Cr measured values using MRI and ^1^H magnetic resonance spectroscopy (MRS) were recently published [32]. Here we correlate the NAA/Cr ratios in different brain regions with monocyte activation and the expansion of subpopulations.

### Flow cytometry studies of monocytes

Peripheral blood was drawn on days −7, 6, 8, and 12 pi, and weekly thereafter. Complete blood counts were obtained using a CBC Hematology Analyzer (Hema-True, HESKA). Flow cytometric analyses were performed with 100 µl samples of blood as previously described [10]. Fluorochrome-conjugated primary antibodies including anti-CD3-FITC (SP34-2), anti-CD4-FITC (L200), anti-CD14-FITC (M5E2), anti-CD16-PE (3G8), anti-HLA-DR-PerCPCy5.5 (G46-6), and isotype control anti-IgG_1_, κ-FITC (MOPC-21) all from BD Pharmingen, anti-CD64-FITC (22) and anti-CD163-FITC (Mac2-48) from Trillium Diagnostics, anti-CD8-PE (DK25; Dako), and anti-CD11b-APC (M1/70.15.11.5; Miltenyi Biotec) were used. Samples were fixed in PBS containing 2% formaldehyde, acquired on a FACSAria cell sorter (Becton-Dickinson) and analyzed with Tree Star Flow Jo version 8.7. Monocytes are first selected based on size and granularity (FSC vs. SSC). From this gate, HLA-DR+ CD14+ monocytes were selected. We note all monocytes analyzed by FSC vs. SSC are HLA-DR+. The absolute number of peripheral blood monocytes was calculated by multiplying the total white blood cell count by the total percentage of each monocyte subset population as determined by flow cytometric analysis.

### Immunohistochemistry

Axillary lymph nodes were collected in 10% neutral buffered formalin, embedded in paraffin, and sectioned at 5 µm. Tissues were deparafinized, rehydrated and incubated with blocking reagents. Newly infiltrating monocyte/macrophages were identified by the expression of myeloid/histiocyte antigen MAC387 (MAC387; Dako) [53]. Mature resident monocyte/macrophage and microglia were assessed using anti-CD68 (KP1; Dako) [54]. Cells that were productively SIV infected were studied using anti-SIV-p28 (MX-0322; Microbix Biosystems) [55]. For quantification, at least 3 non-serial axillary lymph node sections from each of the fourteen macaques were stained for each marker. The number of MAC387+, CD68+, and p28+ cells was counted from 4 arbitrary fields, and the data are expressed as the number of chromogen-positive cells per unit area (mm^2^). Sections were visualized with a Zeiss Axio Imager M1 microscope (Carl Zeiss MicroImaging, Inc.) using a Plan-Apochromat ×20/0.8 Korr objective and analyzed using Adobe Photoshop v4 software.

### *In vitro* infection and minocycline treatment

Peripheral blood mononuclear cells (PBMC) were prepared from EDTA-coagulated blood obtained from healthy animals by Ficoll density gradient separation. CD14+ monocytes were isolated using CD14 MACS microbeads (Miltenyi Biotec). Isolated CD14+ monocytes (>95% purity) were adjusted to a final concentration of 5×10^5^ cells/mL in RPMI 1640 supplemented with 11 g/L sodium pyruvate, 10% fetal bovine serum (Atlas Biologicals), and 10 ng/mL M-CSF (Peprotech Inc). Using M-CSF all monocytes *in vitro* were CD14+CD16+ prior to minocycline treatment. Monocyte/macrophages were infected with a highly macrophage tropic clone SIV316STOP virus (30 ng of SIV p27; a generous gift from Dr. Ronald Desrosiers, NERPC) at 37°C for 24 hours, then washed with PBS containing 2% FBS to remove excess virus. Cells were cultured for 24 or 72 hours with 10 µM and 20 µM minocycline (Sigma Aldrich). Myeloid markers were assessed by flow cytometry using anti-CD14-Pacific Blue (BD Pharmingen; M5E2), anti-CD16-PE, anti-HLA-DR-PerCpCy5.5, and anti-CD163-FITC antibodies. Viability of cells was determined using a LIVE/DEAD Fixable Dead Stain Kit (Invitrogen). Viral replication in conditioned media was quantified by SIV p27 ELISA (Advanced BioScience Laboratories, Inc).

### IL-6 and TNF induction by monocytes *in vitro*

CD14+ monocytes were isolated and cultured as described in the previous section at a concentration of 1×10^6^ cells/mL for 16 hours in non-adherent conditions at 37°C. Monocyte/macrophages were then incubated for 3 hours at 37°C with or without 10 ng/mL or 100 ng/mL LPS (Sigma Aldrich), and 10 µg/mL brefeldin A (Sigma Aldrich) for intracellular detection of cytokines. After stimulation, cells were fixed and permeabilized with BD Cytofix/Cytoperm™ buffer (BD Biosciences) for 20 minutes at 4°C. Cells were washed and incubated with anti-CD16-PeCy7 (3G8) anti-IL-6-PE (MP5-20F3), anti-TNF-APC (MAb11), anti-IgG_1_, κ-APC (MOPC-21), and anti-IgG_2a_, κ -PE (R35–95) all from BD Pharmingen, anti-HLA-DR ECD (Immu-357; Beckman Coulter), and anti-CD14-Pacific Blue antibodies for 30 minutes at room temperature. Viability of cells was determined using a LIVE/DEAD Fixable Dead Stain Kit and dead cells were excluded. Data are expressed as the percent of total monocytes producing IL-6 or TNF.

### Statistical methods

We have previously described kinetics of NAA/Cr over time in different brain regions of minocycline treated versus non-treated animals [32]. Here we used a least-squares means model to identify correlations between our previously determined NAA/Cr in different brain regions and the absolute number of different monocyte subsets. This method allows for the correlation of data points that are not independent of one another, such as repeated measurements of NAA/Cr or monocytes from the same animal. Cross terms between animals and monocytes were performed where a significant cross term indicated that at least one animal's slope ((NAA/Cr) / absolute monocytes) was contrary to a randomly chosen reference animal. If such significance existed, the statistic was considered invalid. Statistical analysis was performed using JMP 7.0 (SAS, Cary, NC). Mann-Whitney U tests were used for all other statistical analyses and performed using Prism version 5.0b (GraphPad Software, Inc., San Diego, CA) software.
